# Researching the performative interface in Rapa Nui: bridging Indigenous knowledges, colonial histories and contemporary performances

**DOI:** 10.3389/frma.2025.1541522

**Published:** 2025-03-21

**Authors:** Moira Fortin Cornejo

**Affiliations:** Ōtākou Whakaihu Waka, The University of Otago, Dunedin, New Zealand

**Keywords:** interface, Rapa Nui performing arts, Talanoa, orality, relationality, dialogue

## Abstract

My research focuses on the performing arts in Rapa Nui. I am interested in performances as ways of navigating and negotiating the complex intersections between traditional/Indigenous and contemporary/Western cultural identities. As a non-Indigenous researcher who has collaborated extensively with the Rapa Nui community, I have had to navigate the interface, learning about and engaging in diverse knowledge systems and methods. The idea of engaging with both Western and Indigenous epistemologies, reflects my belief that both bodies of work can complement one another, and reflects my desire to look at research questions from a range of different angles and perspectives, welcoming and accepting the differences and similarities between worldviews, enriching the possibilities for dialogue between cultures. My research ethos consciously focuses on the positives of cultural dialogue, with a desire to better understand and support intercultural theater practices in Rapa Nui. In my research I have utilized open-ended interviews, framed by Talanoa which is an Indigenous Pacific research methodology which involves deep, open ended discussions and listening, valuing and learning from what is shared in these dialogues and prioritizing relationships between people over rigid, predetermined research agendas. My interactions with the Rapa Nui communities have explored their perceptions of what constitutes “traditional” and contemporary theater/performance practices. The genealogy and influence that “tradition”, as a colonial term, has had over Rapa Nui performing arts over time and space are explored in this article. Colonial histories have effected how contemporary performing arts have been articulated, conceptualized, produced and taught in twenty first century Rapa Nui.

## Introduction: the importance of the performing arts in the cultural interface

In 2008, when I was doing my MA (2010) in Pacific Island Studies at the University of Otago about the history of Rapa Nui Theater, I watched a recording of Tanemahuta Gray's *Maui: One Man against the Gods* (2007). The work of this Māori director excited and inspired me as an example of a “traditional” Māori story which had been staged in a Western and contemporary style—on a stage, with audience, lights, sound, flying trapeze and wireless microphones—without losing the sense that a Māori worldview was at the core of this performance.

The play opens with a stage in darkness; a man and a woman chant the Māori creation story about the separation of Ranginui (Sky-father) and Papatuānuku (Earth-mother). Māori musical instruments resembling the sound of wind play, while four artists engage in an aerial performance. From the center stage, which is covered with fabric, we hear voices naming the eight sons of Ranginui and Papatūānuku, using different tones and intonations, but the same rhythm. Chants begin to crescendo while the fabric begins to rise. Eight actors are revealed; moving forward they repeat a set of movements from a haka, until everyone performs a haka in unison, marking the birth of Ranginui and Papatūānuku's children, and filling the stage with light.

As a performer and actress of Chilean heritage who has lived and performed in Rapa Nui for more than 10 years, and a student of Pacific cultures and epistemologies, *Maui* struck me as very different from the artistic expression of “traditional” Indigenous stories that I had become familiar with while resident in Rapa Nui.

I lived in Rapa Nui, between 1999 and 2012. During this time, I watched and was involved in a variety of unique storytelling forms which were performed not only orally, but also visually and through the body, using takona (body painting), and kaikai (storytelling using string figures as illustration) (Fortin Cornejo, 2023). These performances were based on the style and methodology created by the first Rapa Nui theater troupe founded in 1975, Mata Tu'u Hotu Iti (MTHI). This group was composed of local actors who performed precolonial Rapa Nui oral narratives in different open-air settings on the island, all of which had cultural significance for the Rapa Nui community and many of which linked with origin stories amongst the Indigenous community (Huke, 1995, p. 52). One of MTHI's main performance depicted the arrival of the first Polynesian migration under the command of high chief, ‘ariki Hotu Matu'a. This performance was enacted in Haηa Rau,^1^ the same place where oral narratives tell us that the ‘ariki first arrived on the island. Although the troupe's last performance was in 1999, the work of MTHI is often seen as “traditional” and continues to have a strong and significant influence on the style and content of contemporary theater produced in Rapa Nui.

When I first watched *Maui*, and as I subsequently watched a wide range of Māori and diasporic Samoan theater productions in Aotearoa, it struck me that the blending of “traditional” and contemporary cultural elements appears to be a relatively smooth process, while in Rapa Nui theater this blending of styles is not so apparent. These differences initiated my PhD research journey which began in 2013, considering the ways in which Indigenous epistemologies are being integrated and explored through the performing arts, shaping and influencing the production of contemporary performances that are seen as culturally relevant, reflective, and respectful by Indigenous communities.

Histories of colonization in Aotearoa and Rapa Nui undoubtedly influence both historical and current relationships to theater in these different cultural contexts. Colonialism had worldviews and strategies in common globally, but these of course manifested differently across cultural contexts in interaction with different places, peoples and histories. Colonial discourses and understandings continue to inform current theater practices, and this includes influencing how creativity is valued, and what forms of creativity are seen as possible and acceptable in the present.

In the book *Post-Colonial Transformation* (2001) Ashcroft describes the global impacts of colonialism. He points to the loss of “language,” the way in which “history” is explained and understood by the dominant culture, and the ways in which Indigenous culture has been transformed into an “allegory” (Ashcroft, 2001, pp. 14–15) for the tourist industry. He argues that these factors all influence the creativity of Indigenous communities. In the wake of the collapse of many of the industries and trade agreements established during colonialism, the economies of Pacific Island countries are increasingly dependent on the tourist industry. To meet the demands of the tourist market, the commodification of cultural forms and Indigenous peoples and communities has become widespread. This is especially in the case of Rapa Nui, as performing arts have been transformed, to some extent, into an allegory for the tourist industry. However, performance simultaneously exists with a variety of audiences in mind and narrations of Indigenous stories in Indigenous languages explicitly designed for Indigenous/local audiences have enabled Indigenous theater in both Aotearoa and Rapa Nui to reclaim, acknowledge and extend their particular ways of knowing and being.

Māori theater in Aotearoa has combined elements of tikanga Māori with aspects and techniques from European theater, creating unique blends of “traditional”/Indigenous and contemporary theatrical elements. Tikanga Māori can be understood as referring to “Māori practice or protocol” (Turner, 2002, p. 71) where behaviors, worldviews, and ways of addressing others are strongly linked together. Practicing tikanga Māori defines and produces values and behaviors that Māori communities believe are culturally “ideal, appropriate, [and] correct” (Turner, 2002, p. 75).

In Rapa Nui, theater is firmly grounded in oral narratives and explicitly values “traditional” performances. Since the creation of MTHI, Rapa Nui peoples have continued to perform theater in almost the same way that this group performed it in the 1970s. Their work has become an established standard for creativity. Through theater, Rapa Nui peoples have been able to reflect upon and share both old and new stories, to use theater as a vehicle for exploring and negotiating cultural identities, for expressing desires, frustrations, and humor. Theater has been used as a tool for initiating discussions about culture and identity in Rapa Nui. Theater practice has been further developed within the educational system, where high school students can reflect and contest official colonial narratives, as well as stimulating dialogue with the Chilean community living in Rapa Nui.

My research has consciously endeavored to focus on the positives of cultural dialogue, with a desire to better understand and support intercultural theater practices in Aotearoa and Rapa Nui. By moving past fixed ideas of what constitutes “tradition” and by recognizing the notion of “tradition” as itself of colonial origin, critical reflections on processes of decolonizing creativity may be possible. Cultural heritage, identity, and creativity can be celebrated and explored through theater and the arts. The creative arts can support processes of reflection, generating discussions and dialogues not only about past issues, but also about how societies choose to face the future.

This article begins with a discussion on my positionality as a non-Indigenous researcher working with an Indigenous community. Next, I explore the process of weaving different research methods and how this has affected not only my understanding of Rapa Nui performing arts, but also my relationship with the community. After that I highlight the importance of intercultural dialogue to learn from different ways of knowing and being. Finally I question the weight given to written sources over oral ones. The colonial academic world in which we operate, can often make us forget that there are other ways of seeing and understanding our cultural environment. In this article I conceptualize cultural relations as an exchange, where both sides can contribute and learn. Cultural encounters can create reflective and dialogic interfaces where cultural forms interact with and inform one another creating new cultural forms which reflect “the complex realities of an increasingly globalized and transnational world” (Clery et al., 2015, p. 307).

## Locating the researcher: place, voice, and positionality

My name is Moira Sofía Fortin Cornejo.^2^ I have Indigenous Chilean, French, and Spanish heritage. I was born in Santiago, Chile. I have lived, studied, worked, and performed in Chile, Germany, Rapa Nui, and Aotearoa. I come from a country which is classified as a “third world country” but which is also, in Rapa Nui, a colonial power. My country of birth was colonized a long time ago by the Spanish who significantly affected the lives of all the different Indigenous cultures living in the territory. During my childhood I studied at a bicultural German school, and this significantly influenced my cultural landscape. By the age of eighteen I spoke fluent German and knew more about German history and geography than Chilean, even though the school aimed to celebrate both cultures. Now in my adulthood I speak fluent English, and my command of the French language is intermediate.

The ability to speak four languages has enabled me to communicate with people from different cultures; allowing me to share my artistic experiences as a local from Santiago, Rapa Nui and Aotearoa with other artists from these locations, who in return have shared with me their stories, their protocols, their dances and songs, as well as their languages. I have enduring family connections in Rapa Nui. My grandmother Berta Alfaro Rojas chose to develop relationships with people from Rapa Nui in the 1970s. Since then, my family has been connected to the Rapa Nui culture and its people. My interest in Rapa Nui culture was passed on to me from her daughter, my aunt, Eliana Cornejo Alfaro, who is married to a Rapa Nui man, Juan Hey Riroroko, and lives in Rapa Nui until the present day.

Living and performing in Rapa Nui for more than ten years I have been working with artists who share a common creative passion for the performing arts. Cultural encounters through the creative arts can be characterized as a collaboration, in which both parties work together. Although it is important to recognize the enduring impacts of colonialism, and unequal power relationships which extend into the present; I argue that the creative arts, and particularly theater, can enable people to explore and to challenge the typical colonial relationship between the oppressor and the oppressed, and to transcend it. Reciprocity, or hakakio in the Rapa Nui language (Te Re'o), is at the heart of any relationship. Reciprocity entails collaboration, negotiation, and sharing between cultures. It involves an equal process of give and take, involving a commitment to return what has been taken from another person over time and through ongoing relationships, but not as the appropriation of cultural products. Respect, mo'a in Te Re'o, is another core element in every cultural encounter. There are various ways in which the researcher can be respectful to the communities they are researching with. The most important one is to acknowledge their contribution to the research, not only by citing them, but also by recognizing the value of their guidance, knowing that without their knowledge the research would not have been possible.

Throughout my research journey Māori, Samoan, Rapa Nui, Pākehā, and Chilean theater practitioners shared ideas and knowledge with me. Each cultural group can potentially benefit from my research; they can use it, change it, or discard it completely, it is up to the reader. In terms of my own research and performative practices, the interface that researching the performing arts provides, has enabled me to incorporate culturally appropriate methodologies and knowledge systems into my work. This includes rehearsing a play that comes from a colonial background and/or conducting research with non-Indigenous performance groups. My experience researching the performative interface has also enabled me to share knowledge of decolonised theater practices with practitioners from the English, Spanish and French speaking worlds. Above all, my research has strengthened my relationship with the Rapa Nui community by sharing approaches learnt with Rapa Nui artists, but also by consulting and sharing my findings with the Rapa Nui community.

## Weaving research methodologies at the interface

The research methods used in most of my research projects combine Talanoa (Vaioleti, 2006), community-based participatory action research (Getty, 2010), insurgent research methodology (Gaudry, 2011) and contain an awareness of my role as a liminal (Bendrups, 2000) and Multi Perspective Culturally Responsive (MPCR) researcher (Clery et al., 2015). The combination of these methods reflects my belief that Western research methods are not the only valid ways of gaining knowledge. Indigenous methodologies and protocols are appropriate research methods within indigenous communities. These different research methods complement each other and reflect my desire to embrace and accept differences and similarities between worldviews, fostering intercultural dialogue. Researching on Rapa Nui performing arts has been informed by employing this mix of Western and Indigenous methods in the different fieldworks undertaken, which have been further augmented by other research methods including semi-structured interviews, library research including textual study, discussing the different existing versions of the oral narratives performed, watching filmed performances, and my own experience performing within Rapa Nui artistic frameworks.

I became a permanent resident in Rapa Nui in 1999. I started working as a drama teacher at the only school on the island at that time, Liceo Lorenzo Baeza Vega, where I worked until 2004. In this role I attempted to introduce students to theater pieces written by authors such as Shakespeare, Moliere, Pirandello, and to the work of well-known contemporary Chilean playwrights, such as Radrigán and Griffero. My idea, perhaps as a naïve and recent graduate from drama school in Chile, was to use these authors as tools to encourage different forms of expression in the students. At that time, I thought that living on such a tiny island it could be beneficial for the students and the audiences to know and understand different realities and ways of living and speaking through theater. I hoped that culturally diverse and different performances might help audiences to reflect on their own understandings and behaviors through reading or performing different types of drama pieces. However, these workshops generally failed to engage students' interest or imagination, mainly due to the fact that foreign material did not seem at all relevant to their lives.

This experience taught me that I had to decolonize my own way of teaching and practicing theater. Secondly, it encouraged me to acknowledge that work and research involving Indigenous peoples should consider the outcomes of the research, and how they might be of benefit to the wider community (Smith, 1999, p. 191). Although Western researchers may try to utilize post-colonial methodologies and theories “to deconstruct the influence of colonialism, it does not [always produce research that] reflect[s] Indigenous ways of knowing” (Getty, 2010, p. 7). In fact, research using a postcolonial perspective could primarily reflect the values of the researcher (Getty, 2010, p. 7), rather than the values of the community. Often the community does “not see themselves represented in texts or if they do see themselves, they often do not recognize the representation” (Wilson, 2001, p. 216). To avoid this disconnection with the participants and communities involved in this research, I decided to use methodologies that I consider more appropriate to performance research which seeks to be of use to Indigenous communities, and to engage with them.

Pacific researchers have developed research methodologies which seek to reflect and respect the protocols of Pacific cultural contexts. Talanoa is a Pacific research method which enables a “personal encounter where people story their issues, their realities and aspirations” (Vaioleti, 2006, p. 21). Talanoa allows people to participate in an open conversation which is not set or pre-determined in terms of its content, engaging participants in an oral and collaborative critical discussion which can generate knowledge on a range of different topics. Violeti explains that Pacific ways of gathering information and creating knowledge are spoken interactions which are based on verbal cooperation. In using Talanoa as a research method it is very important to pay attention to the way in which people talk about their lives, especially to the language they use and the associations they make, which can help to reveal their worldviews (Gilligan 1982, in Vaioleti, 2006, p. 25). This approach is a profoundly relational and dialogic approach.

While conducting field work for my MA research in 2009 in Rapa Nui, focusing on the history of Rapa Nui theater practice, I looked for culturally-situated research methodologies in use on the island, and discovered that although no Rapa Nui specific research methods have yet been conceptualized, the procedure in which the meetings with participants were held, related well to the ideas and protocols underpinning Talanoa as a research method. Talanoa seeks to create relationships between people, and to minimize the space between the researcher and the research participants, giving all participants in the dialogue the opportunity to be reflected, and to feel related to the research in which they are participating (Vaioleti, 2006, p. 25). Talanoa is a culturally-situated Pacific research method which is “context specific and responsive to the needs of people in a given situation” (Clery, 2014, p. 108).

Community-based research methodology and insurgent research methods both focus on using an Indigenous world-view, and on putting the community at the center of the research to help ensure that the results of the research can be used by Indigenous communities, if they wish to do so. Insurgent research methods argue that “the Indigenous community are the final judges of the validity and effectiveness” (Gaudry, 2011, p. 117) of the research. In addition, Gaudry states that insurgent research is “action oriented and works as a motivating factor for practical and direct action among Indigenous people and in Indigenous communities” (Gaudry, 2011, p. 117). In November 2024 I traveled back to Rapa Nui, to find that the educational community is utilizing theater practice as an important educational tool to decolonize education and to promote the use of the language by dramatizing their stories. During this time I could engage in conversation with former colleagues at the different schools regarding the implementation of these dramatizations was a way of minimizing “colonial interference” (Gaudry, 2011, p. 114) within Indigenous educational communities.

As someone who has substantial lived experience in Rapa Nui, but who was born in Chile, my status as a researcher is complex. I am not an “insider” in the way that an Indigenous Rapa Nui researcher would be. However, my personal and professional experiences in Rapa Nui and my relationships with people have combined to make me comfortable and familiar with Rapa Nui patterns of conversation and culturally-situated ways of building rapport. As a result of my experiences in the performing arts and of my intimate involvement with the performing arts community on the island I am an “insider” to this particular research topic in some ways.

To help articulate my relationship to the communities I researched with, I conceptualize myself as a Multi Perspective Culturally Responsive (MPCR) researcher. A MPCR is someone with “multiple and hybrid cultural positioning” (Clery et al., 2015, p. 313), who has cultural and linguistic fluency that is based on lived experience in a variety of places and a strong sense of belonging to a range of diverse communities. MPCR researchers are not completely “insiders” or “outsiders” to their research communities but can instead be thought of as researchers who inhabit liminal spaces “…on the threshold between one world and another” (Ven Gennep, 1965, p. 22) “work[ing] within complex and fluid relational continuums” (Clery et al., 2015, p. 303).

Clery et al. (2015) point out that MPCR researchers “belong” in and are attached to many places. “MPCR researchers have lived experience in a variety of different spaces, places, cultures, religions, and languages” (Clery et al., 2015, p. 315). Using these lived experiences and levels of cultural awareness and fluency as the basis of their relational approach to working with people “MPCR researches [seek to] work toward achieving mutually empathic relationships with Indigenous communities” (Clery et al., 2015, p. 316). Relationships grounded in mutual empathy are demonstrated when:

[T]he community feels that the researchers have a good understanding of local cultures, and that they have demonstrated their commitment to the wellbeing of Indigenous peoples (Clery et al., 2015, p. 316).

MPCR researchers focus on engaging, connecting, and collaborating with communities allowing researchers to be “reflexive and critical without being divisive or excluding researchers or participants on the basis of their race, ethnicity, or location” (Clery et al., 2015, p. 316). This methodology supports the conceptual framework used within my research in which different ways of understanding, knowing and seeing can collaborate and dialogue together for a better understanding of different realities, perceptions, and points of view regarding Rapa Nui theater practice. For example, Rapa Nui performances are mostly in their entirety spoken in the Rapa Nui language. As a non-Rapa Nui researcher, this encouraged me to learn the language as much as possible. But not only the spoken/verbal language, but also visual language, including symbolic movements danced and/or designs used on stage, or painted on the performer's body. These symbols if known are silent texts enabling audiences to identify the charater's affiliation to a specific clan or area of the island, and therefore understand their intentions within the play.

The liminal researcher methodology was conceptualized by Daniel Bendrups in his study of Latin American migrant music in Australia, and it complements the concept of the MPCR researcher. Bendrups reflects on the ethical aspects of fieldwork, and especially on the role of the researcher. According to Bendrups (2000) “fieldwork should be conducted from a liminal perspective, that is, a position at the point where [your] own culture and the culture of [the] research subject intersect” (p. 35). The success of this methodology depends fundamentally on the researcher's “ability to interact with other cultures” (Bendrups, 2000, p. 35). This interaction between cultures can be supported by community-based research because it seeks to acknowledge Indigenous worldviews throughout the research process. Since the word theater does not exist in Te Re'o, I had to find the Rapa Nui concept for narrating a story in front of an audience at a specific time and place. The general word used is ‘a'amu, and depending whether it is an old or a new story the concept becomes ‘a'amu tuai and ‘a'amu ‘āpı̄ respectively, becoming the notions used throughout my research.

The viewpoints of Indigenous performers and artists are at the center of my research. Through dialogue I aim to ensure that their voices and opinions are strongly present. The research methods I have chosen to draw from and to combine support and enable Indigenous people to be actively involved in the construction of knowledge and in the creation of representations about them through “participat[ing] as researchers in partnership with academic researchers” (Getty, 2010, p. 5). I have given participants the “chance to state and restate [the] meanings… and to modify, delete, and adapt [the] meanings according to local customs” (Bishop, 1998, p. 206). The concepts of the liminal researcher, the MPCR researcher, and the approaches used in community-based research relate back to Talanoa because they allow collaborations based on partnership, reciprocity, and respect on an equal basis. In this case both the researcher and Indigenous peoples can ask questions, to challenge different opinions, and to find possible solutions to different issues. When working on the manuscript of my book *Rapa Nui Theater: Staging Indigenous Identities in Easter Island* (2023) I sent to each contributor a section of the text where they had collaborated. I asked them to read it and to add, modify or delete anything they saw fit. Some contributors did want to make changes to their texts, updating and strengthening their ideas and perspectives as well as validating my work in the process.

Community-based research and insurgent research methods speak of collaborating with Indigenous communities and putting them at the heart of research. In this same spirit I have engaged in a variety of activities with different Rapa Nui groups and institutions, transmitting my research findings as they emerged during my fieldwork. These activities included a public presentation of my research at the local museum, participation in a radio program about theater where people could call in and ask questions, collaboration in a storytelling workshop with children from the community and publishing an article in the local magazine. In addition, I worked in collaboration with the Cultural Corporation of Easter Island, a department within the Rapa Nui City Council whose main responsibility is overseeing the various research projects taking place in the island.

Another Indigenous method present in my research is Konai Helu Thaman's kakala methodology. I came across this method while searching for a way of sharing my research with the people of Rapa Nui. According to Vaioleti (2006) kakala is a Pacific concept of teaching and learning, in which the term kakala refers to the process of creating and gifting a garland of fragrant flowers and leaves that have been woven together to honor a person or project. In most Pacific cultures, there is a special tradition and protocol associated with kakala. Keeping this process in mind throughout my research, that is, consciously deciding how I will be sharing my research with the Rapa Nui community, enables me to keep the Rapa Nui community center and front of my research, as I am in constant communication with them, seeking their guidance, presenting preliminary findings for their feedback as well as sharing links to published research.

Helu Thaman (in Fua, 2006) explains that in the making of kakala (tui kakala) three different processes are involved, which are toli, tui, and luva. Firstly, toli involves not only deciding, selecting and picking the different flowers and leaves required for making the kakala, but also ranking and arranging them according to their importance (Fua, 2006). In this case the flowers and leaves of my kakala were the participants with whom I would be discussing a variety of topics regarding Rapa Nui performing arts, the performances recorded, the oral narratives chosen as the more representative texts of Rapa Nui repertoire for its analysis, as well as the written sources used to build up the bibliography.

Secondly, tui is a vital step in the process of weaving the kakala, selecting, sorting, grouping and arranging the flowers and leaves before weaving the kakala for a particular person or purpose (Fua, 2006). In order to explain to a Western readership how Rapa Nui theater was composed and performed I had to de-construct all the different elements that can be classified as Rapa Nui performing arts, so the reader could understand what was meant by hoko (dance) or riu (song), for instance. After the de-construction was done, without losing the essence of each element, the re-construction of these elements began, outlining a distinct and unique type of Rapa Nui performance called ‘a'amu tuai which is the dramatization of oral narratives (Fortin Cornejo, 2023).

A deconstruction demonstrates that beneath the clam surface of unity a thing puts forth there lies a multiplicity of competing elements… A deconstruction shows that things are never as simple as they seem, never as easy as they look, never as finished as they make themselves out (Caputo and Cook, 1995, p. 13-14)

Finally, luva is the giving away of the kakala to the wearer, who might be a dancer, a special guest or someone leaving on a long trip (Fua, 2006). The term refers to the stage when the research about a community is given back to the community, to acknowledge their participation in and ownership of the knowledge that has been woven together, gifting it back to communities so that others can benefit from it. Kakala methodology is carefully designed according to a specific purpose, which “provides a framework of data collection through toli, data processing through tui, and application of knowledge gained through luva” (Fua, 2006, p. 4). The implementation of that knowledge is an important part in the context of Polynesian values of love or koa in Te Re'o, respect or mo'a and reciprocity for each other known as hakakio in the Rapa Nui language.

## Intercultural dialogue through the performing arts

My research on Rapa Nui performing arts has explored the construction of the “traditional” and the contemporary, and the implications and consequences that the understandings of these concepts may have for cultural creativity. The term “tradition” is of relatively recent historic origin. Its contemporary use suggests a continuity with the historic past, which is not always accurate (Hobsbawm and Ranger, 2012). “Tradition” is always constructed through the lens and values of the present. Pacific anthropologists have debated how much weight should be given to the colonial meaning of “tradition” (Jolly and Thomas, 1992). Their work has acknowledged the diverse meanings of “tradition” found across Pacific Island languages and worldviews, thus initiating processes of interrogating the term “tradition.” Through my research I have presented several examples of Māori, Samoan, and Rapa Nui theater that demonstrate different perceptions of what constitutes “tradition.”

In present day Rapa Nui, the colonially inherited idea of preserving “traditional” aspects of culture is enduringly powerful. Theater productions which deal with “traditional” themes in a “traditional” style are highly valued among the Indigenous community. In contrast contemporary theater and the arts are often seen as betraying “tradition” and culture. While the reproduction of “traditional” styles and stories has been a way of celebrating the survival of Indigenous cultures in the face of colonial hegemony and domination, these polarized understandings could significantly restrict creative possibilities and could themselves be understood as extensions of colonial dialogues which perceived Indigenous cultures as “dying” (Urry, 1979, p. 15), focussing on the preservation of “tradition.”

Hobsbawm and Ranger define the term “tradition” as a set of activities that seeks to instruct values and norms of behavior by repeating them over time, implying a continuity with the past (Hobsbawm and Ranger, 2012, p. 1). The terms “tradition” and “traditional” are conceptualized within my work as ideational and cultural platforms which are inextricably connected to the creation of contemporary works. “Traditional” practices necessarily and paradoxically always involve contemporary cultural elements; they are constantly re-created and re-interpreted in and through perspectives and values from the present.

Through my research I have explored complex and contested concepts including bicultural theater, to understand theater as a liminal space where different cultures meet and dialogue. Bicultural theater was developed in Aotearoa as a response to wider political and conceptual movements in the country, aiming to give equal respect and inclusion to Māori and Pākehā (European settlers) cultures and worldviews. Bicultural theater is defined as theater that arises “in response to the influence from both Māori and Pākehā cultures” (Greenwood, 2002, p. 57). It uses these different cultural perspectives and influences in the process of conceptualization and rehearsal, as well as integrating them into performances. Greenwood's definition of bicultural theater acknowledges the impacts of cultural influences in the process of creating theater. This approach could also be applied in the Rapa Nui context, supporting the creation of contemporary works that are grounded in culture, rather than defined by fixed ideas of “tradition.” However, I argue that in an increasing globalized world, perhaps bicultural theater is not an applicable theoretical framework for understanding and conceptualizing artistic expressions in the twenty-first century. To understand creativity today the concept of multicultural theater may be more appropriate. This is theater that arises from contact between multiple cultures, including other Pacific Island and Asian cultural groups connecting through social media and festivals across the world.

Cultural encounters through theater “inevitably entail a process of encounter and negotiation between different cultural sensibilities” (Lo and Gilbert, 2002, p. 31), therefore focusing on processes and possibilities for “intercultural exchanges within theater practice” (Pavis, 1996, p. 1). Intercultural theater is interested in the overlaps and intersections between different cultural forms. Working in the performative interface means that I am consciously focusing on the marginal spaces, where different “traditions” interact with and influence one another, and in which new cultural forms and expressions are created. For example in my research about the introduction of non-Indigenous classical instruments by music school Toki in Rapa Nui, I discovered how the teaching of these instruments has influenced the musicalization of plays. In 2018 the soundtrack of the production *Vakaroa* (Liceo Aldea Educativa, 2018) was created using piano and cello in conjunction with instruments considered typical of Rapa Nui such as the guitar and the bass drum. This development was new to the performative tradition in Rapa Nui, but equally valid as the lessons from Toki are extrapolated to other areas of the Rapa Nui performing arts by its students.

Over time the creation of the contemporary performing arts has been influenced not only by histories of colonialism but also by the ongoing pressures of the globalized world such as the tourist market. Performances and festivals produced to satisfy the tourist gaze “are positioned as signifiers of past events, epochs or ways of life” (Taylor, 2001, p. 9) linking authenticity closely to the past “original” version. However, ideas of what is “traditional” or “authentic” may change depending on the viewer's background, therefore changing perceptions of what constitutes “tradition” and “authenticity.” Indeed, ideas of “tradition” and “authenticity” are contested, negotiated and re-negotiated through different versions, perspectives, and interpretations.

The production of contemporary performances involves a series of local and global negotiations and articulations between “multiple parts and joined together through complex political, social, and cultural processes” (Powell, 2020, p. 128). Teaiwa (2017) conceptualized this articulation “as a metaphorical train with carriages” (p. 5), “an articulated limb” (p. 66) or “the form of the connection” (Grossberg, 1986, p. 53) between the ever-present colonial power, the growing demands of the tourist industry and the preservation and revitalization of the Rapa Nui culture through the performing arts. In this sense, the Rapa Nui performing arts are a cultural articulation, with the potential to change, negotiate relations with the colonial power or expose the inequalities and injustices suffered by the Indigenous population forcing the relationship to be disarticulated, temporarily or permanently (Powell, 2020, p. 128).

Grossberg (1986) asks us to consider “under what circumstances can a connection be forged or made?” (p. 53). The articulation of contemporary ‘a'amu tuai, was created and led by the group Mata Tu'u Hotu Iti in 1974 responding to the ongoing injustices and restrictions the Chilean colonial powers imposed on to the Rapa Nui people.

This troupe endeavored to show, reclaim, revive and to celebrate Rapa Nui's precolonial ways of knowing and being. They aimed to reflect the Rapa Nui reality, culture, and sensibility, which involved a complex process of cultural disarticulation which was “inevitably violent and traumatizing” (Teaiwa, 2017, p. 66). This cultural disarticulation process entailed moving away from the imposed Chilean colonial culture that systematically ignored Indigenous Rapa Nui forms of expression. This process required the Rapa Nui community to re-articulate the way in which they wanted to express their point of view about Rapa Nui history.

Throughout its history Mata Tu'u Hotu Iti performed different oral narratives in specific areas on the island and it was commonly understood that every performance had its historically specific or significant place. Following in the footsteps of their ancestors, MTHI used their knowledge of historical performances that had been produced in specific places around the island, conceptualizing the Rapa Nui landscape itself as a stage with deep cultural resonance. Places are embedded with cultural meanings and significance for Rapa Nui people. Some sites evoke particular pieces of Rapa Nui history. This was the reason why the troupe chose a specific location for each performance depending on the type of narrative to be performed.

The tā-vā theory of reality by Tongan scholar Māhina (2010) argues that in Indigenous Pacific epistemology tā (time) and vā (space) are inseparable and can be transcended through our relationships to each other and deep connection to the cyclical rhythms of life. To gain a deeper understanding of natural, conceptual, and sociocultural concepts and practices Ka'ili et al. (2017) argued that concepts of tā and vā must be examined in relationship to one another. Pacific ways of conceptualizing tā and vā are understood through the treatment of the past, present, and future in Indigenous epistemologies (Ka'ili et al., 2017, p. 4), where the past and the future are constantly mediated in the ever-transforming present (Hau'ofa, 2000). Constructions of temporality in Oceania value “not only understanding what came before (genealogies) but also the shifting meaning of those legacies, their recurrence and invocation, and their bearing on potential futures” (Powell, 2020, p. 124). Following the work of Māhina (2010), I argue that the performance of ‘a'amu tuai is a physical manifestation of genealogy of time and space in Rapa Nui, representing physical, emotional and social arrangements which are negotiated through performance. ‘A'amu tuai can also be understood as a physical manifestation of spatial relationships which consciously interact with the physical environment over time (Van der Ryn, 2017).

For example, the troupe addressed the genealogy of these performance spaces by selecting various locations where the Rapa Nui peoples had previously commemorated events in the community, ensuring that the meanings, importance and solemnity of particular locations gave additional cultural and historical significance to contemporary performances happening there. These places intersected “temporal-spatial… human phenomenon, across nature, mind and society” (Māhina, 2010, p. 168). These significant locations acted as physical and cultural reservoirs of knowledge, restoring in some ways aspects of pre-colonial time and space as past practices and stories were enacted there (Andrade, 2004).

It could be argued that the selected places were those that had mana, which is a symbolic or representative principle in various Oceanic cultures. In this case, mana is not necessarily understood as power, nor associated with politics, but rather “it is understood in terms like repetition, balance and complementarity” (Tomlinson and Kāwika Tengan, 2016). For example, when the use of a particular space, such as Haηa Rau, known as Anakena beach, is repeated over time to perform the story of the arrival of the first Polynesian migration that is commonly understood to have occurred there in the past, the entire place acquires mnemonic characteristics, helping communities to remember history and maintaining genealogical links connecting the past, present and future. Mana is encountered and mediated through a particular ritualized use of space (Georgina, 2017) within the production of ‘a'amu tuai.

In this context and responding to colonial pressures, ‘a'amu tuai/‘āpı̄ is also the ultimate venue for the expression, performance, recreation and reclamation of Rapa Nui Indigenous identity and culture. Culture is defined here as comprised of the best forms of human activity that endure over time and space (Māhina, 2010). As a project involving cultural articulation, created in the intersection of historical and colonial social activity, the stories and performances produced by Mata Tu'u Hotu Iti as well as the methodology used to produce each performance, have lasted over time and space. In this sense, ‘a'amu tuai has been an arena from which Rapa Nui peoples have been able to creatively emphasize and reassert their Oceanic identity, and to distance themselves from Chilean colonial culture. For example in 2015 the production *Hoa Haka Nana'Ia* (Liceo Aldea Educativa, 2015) is an open demand to the British Museum to return the moai (stone statue) placed in its permanent collection. This ‘a'amu ‘āpı̄ supports the global movement of repatriation of cultural artifacts taken during colonization.

## Orality, relationships and communal memory over written sources

Rapa Nui performing arts have a long history which is embedded in culture. Although the word “theatre” does not exist in the Rapa Nui language, the concept of performance and the notion of theater, that is, to perform a story in a specific place, at a specific time to an audience, is very much alive in contemporary Rapa Nui society. When I embarked on my MA research project, the initial main question was: how to discuss and analyse an art form that looked like theater from a western perspective but was not defined as such in the Indigenous language?

Establishing the definition of Rapa Nui theater, known as ‘a'amu tuai, has been crucial “to assists [non-Rapa Nui] readers to thinking about theater beyond the rigid disciplinary and colonialist confines that often structure its study in institutional settings” (Etherington, 2024, ADS Review). A reoccurring question I receive when sharing my research with the Western academic community is “Where is the cientific evidence that supports these oral narratives?” and whether it would be “possible to read the texts of these performances?”. My answer to that is that in Rapa Nui Theater there is no text, if anything at all, there is a one-page summary with the main action points. Rapa Nui theater is oral in its inception. Rapa Nui Anthropologist Paloma Huke highlights that the members of MTHI did not have formal or western theater training. This is seen as a positive aspect of their work, as it gave performers freedom to devise pieces inspired by “anecdotes and events” (Huke, 1995, p. 44), which were orally narrated by the older members of the MTHI group.

Theater companies in the Pacific produce performances which can be described with the western concept of community theater. The ideas expressed through these performances are shared in close relationship among both actors and audience members. An interesting aspect of community theater is that it highlights the true sense of theater: its relationship with the audience. Theater creates a relationship between audience and actor (Grotowski, 2002, p. 20), because it is an event we live with others (Dubatti, 2011). Reason (2004) explains three characteristics that occur when people talk about the experience of having participated in a live event, like theater: the importance of shared memory, the awareness of the performer's humanity, and the shared experience with other members of the audience. These characteristics of theater is what Dubatti calls “convivio” (Dubatti, 2011, p. 35). From the theatrical encounter, and through dialogue between actors and spectators, convivio multiplies the activity of giving and receiving (Dubatti, 2011, p. 36). A characteristic of community theater is that it is a social engagement, in which the activity of giving and receiving is a continuum. In community theater, this reciprocal continuum happens not only during the performance, but social engagement also occurs during the creative process, because “the aesthetics of community theater are shaped by the culture of its audience” (Lo and Gilbert, 2002, p. 14).

MTHI productions are known as ‘*a'amu tuai*, which literally translates as old narratives (Fortin Cornejo, 2023), demonstrating that MTHI understood theater as an extension of storytelling. ‘A'amu tuai are full of abstractions, frequently relying on symbols, exaggerations and a deep and descriptive narrative, which, due to its historical nature, is an important document for future generations since they are considered true accounts of the history of Rapa Nui and are transmitted from generation to generation as true epics (Huke, 1995) orally and in the Rapa Nui language.

As previously explained the Pacific way of sharing knowledge is oral. To me, it's not only important to pay attention to the way in which people talk, but also to acknowledge the language in which they chose to communicate. Although I am not fluent in Te Re'o Rapa Nui, the Indigenous language, while living in Rapa Nui I understood and regularly participated in conversations held in Te Re'o. Indeed, during my fieldwork for my MA in 2008 some participants engaged in conversations entirely in Te Re'o (Fortin, 2010).

Language is of great importance to cultural understanding, and to conducting successful research with Indigenous communities. As non-indigenous researchers, when collaborating with Indigenous communities we must not assume that Indigenous communities are fluent in a colonial language, either English, Spanish or French. Even if they are fluent in a colonial language, concepts and understandings may not be translatable, so the explanation of Indigenous world views in colonial languages may lack depth. While translation implies the meanings are similar, the sound, grammatical order and syntax of the translation is different, opening the meaning to another aspect of the same topic.

When I write, I try as much as the publisher allows me, not to italicize Indigenous and foreign words. Placing languages other than English in italics degrades and contributes to the “othering” of languages. The conceptualization of knowledge in my work is inclusive, acknowledging different languages, perspectives, and worldviews. This decision is reflective of my desire to allow different culturally-situated readers to connect with this research in different ways.

The ability to read four languages has enabled me to bring together a wide range of literature, as a way of reflecting the diverse linguistic realities across the Pacific. Most of the literature about the historical production and influences of the performing arts in Polynesia has been written in English, mostly by non-Indigenous researchers, especially the accounts written in the nineteenth and twentieth centuries. This is the case for ethnographers who visited Rapa Nui in the early twentieth century. Texts such as *The Mystery of Easter Island* by Katherine Routledge (Routledge, 2007), *Ethnology of Easter Island* by Alfred Métraux (Métraux, 1971), *Los Misterios de Isla de Pascua* and *La Tierra de Hotu Matu'a* by the missionaries Father Bienvenido de Estella (de Estella, 2007) and Sebastian Englert (Englert, 2007) respectively, have been great sources of information regarding Rapa Nui history and customs to date.

Apart from these written sources, I am always including work written by Rapa Nui themselves, especially dissertations written by Rapa Nui students in several Universities in Chile. For example, Rodrigo Paoa and his dissertation called “La Recreación en Isla de Pascua” written in 1983 and the book “Mata Tu'u Hotu Iti” written by Rapa Nui anthropologist Paloma Huke in 1995. Rapa Nui teacher Virginia Haoa Cardinali and Rapa Nui historian Cristián Moreno Pakarati have both written extensively about the consequences of colonialism on education and on the Rapa Nui society respectively. There is also another body of work that it is worth mentioning, which is the work written by Chilean scholars, whose work has been validated by the Rapa Nui community, through the long-lasting collaborative relationship that these scholars have with the Rapa Nui community, this includes myself. Most of these scholars have been living in Rapa Nui for the last twenty or thirty years.

Written sources are highly valued in academia, and the weight given to these spruces comes from colonial times. The impact of missionary work and the imposition of literacy on Indigenous communities has been widely studied. The act of teaching oral communities to read and write meant a “reordering of the mind” (Herbert, 1991, p. 167), and “probably changing the thought patterns in them” (Topping, 1987, p. 52) resulting in “the domestication of the savage mind” (Goody, 1977). Later, another critical dimension was added “the printed word” (Topping, 1987, p. 49) which helped colonial powers in rearticulating a new hegemonic process, introducing a new type of authority to the islands, fostering the colonial written word as authoritarian and superior.

This shift from oral to literate societies contributed significantly to the erosion of precolonial languages and cultures. Over time the expansion of literacy through school systems resulted in the increasing reliance on the written word as opposed to orality and/or memory. The imposition of this type of learning brought sociopolitical and cognitive consequences. Schools assumed the responsibility of preparing people to live within those Western institutional frameworks. The learning experience of children who based and developed their learning through oral language activities done collectively were discarded and replaced by completely different educational patterns and activities done individually and in isolation (Topping, 1987).

For oral communities, languages not only refer to forms of verbal communication, but they also reflect culturally situated worldviews, and ways of thinking (Cook and Bassetti, 2011, p. 80). In the Rapa Nui culture songs and dances are strongly interconnected to oral narratives and therefore to language, reflecting ways of transmitting knowledge in precolonial times where information must have been recited or sung (Fortin Cornejo, 2023). However, and although “oral cultures produce powerful verbal performances [this] may in fact no longer be possible once writing has become entrenched in a culture” (Gee, 1986, p. 725).

From my own experience living in Rapa Nui, it is important to recognize that narratives transmitted orally in Rapa Nui have infinite versions that might vary from family to family and that truth is contingent upon understanding the viewpoint of the speaker. The maintenance of oral narratives necessitates ongoing performative interactions between Rapa Nui peoples. However, the written versions of some of these oral narratives are the main traces of what it is left from precolonial Rapa Nui culture as oral cultures have been decimated over time. As the world changes so do forms of verbal or visual communication that are being discarded by more modern and permanent forms of communication. Thane (1999) explains that moving from oral to literate is a significant shift, impacting the use of memory as individuals and as a collective. When researching on the use of theater to promote orality in younger generations, it became clear that after literacy was imposed in Rapa Nui individual memory “came to be less important to society and less valued” (Thane, 1999, p. 161). Thane questions if the term “memory” is accurate or even useful to the idea of a common past where “the interpretation of the past is accepted by a whole community” (Thane, 1999, pp. 166–167).

The genealogical and relational approach to knowledge transmission has been a conscious methodology used by MTHI. The erasure of oral cultures has been challenged by ongoing production of ‘a'amu tuai (Rapa Nui theater), especially the work done by high school Aldea Educativa, is crucial in maintaining and integrating a variety of forms of Rapa Nui oral narrative and artistic expressions. It is fundamental and essential as it shows how collective memory in this community “is supported through a mosaic of different forms” (Wareham, 2002, p. 196) where dances, songs, kaikai (string figures), takona (body painting) complement the spoken narration of the story, by singing or showing through gestures or symbolic movements other aspects of the story being told.

The transference of collective memories in many Pacific cultures was conducted orally through generations “rather than captured in record form” (Wareham, 2002, p. 194). Although Rapa Nui possessed a unique writing system, called rongorongo, written records that were created by missionaries through a Western lens were firstly “mired in their own expectations and beliefs, and usually misinterpreted or ignored the perspectives, events, and stories of islanders” (Wareham, 2002, p. 194). The dominant historical understanding is that the “voluminous writings” are valuable documents that made available “details about the many peoples they encountered, making them crucial for recording indigenous pasts” (Grimshaw and May, 2010, p. 1), it is important to think about the voices and perspectives that are silenced through a reliance on these dominant written accounts that “exclude… large segment[s] of Pacific history from the entire precontact period” (Wareham, 2002, p. 194). The reality is that the “small proportion of actions” recorded by missionaries in fact only offer “at best a sliver of a sliver of a sliver of any such possible memory” (Harris, 1997, p. 137).

I argue that through the production of theater and the constant practice of Rapa Nui performing arts, capacities for orality and for remembering through sharing oral cultural forms which were so present in Pacific cultures before the arrival of the missionaries, can be revitalized and empowered. ‘A'amu tuai has proved to be highly successful in terms of reflecting an Indigenous Rapa Nui world view. It became so successful that this style of theater has become broadly accepted as reflecting precolonial ways of knowing and being, and it has become one of the foundations in which performing arts are taught, practiced and understood in contemporary Rapa Nui (Fortin Cornejo, 2023).

The written or printed word undermined the practice and transmission of oral cultures, relegating the spoken word to the world of the ephemeral where it does not last over time (Topping, 1987). In this regard the Māori scholar Sir Peter Buck states:

Civilized man has become more and more accustomed to learning with the eyes and less and less with the ear. The eye of the civilized man depends on notes and books. The ear of the uncivilized man has to depend on memory. As the taking of notes increases under our modern education systems, so the cultivation of memory decreases (Buck, 1926, p. 187).

Not only the ability to memorize decreases, but the ability to orally narrate a story to an audience. The connection with the social groups also decreases. This has lasting consequences in contemporary Rapa Nui because communal spaces for the performance of oral cultures and for community interaction with ancestral stories has also decreased.

The ability to memorize a story is not necessarily related to the ability to tell the “correct” version of events. The version we give authority to and memorize will depend on the source from which we learned it and will reflect historical power relationships and those influencing the contemporary moment of performance. An awareness of how dominant voices and historical power imbalances create, and privilege certain versions of stories creates space for other versions to coexist without being judged as erroneous or lacking authenticity.

The production of theater in Rapa Nui has been an outstanding way of fostering cultures of orality in the twenty-first century. Theater has been used as a tool for educational purposes, relating to the use of Indigenous languages and using theater as a tool for Indigenous communities to orally express creators” own versions of historical events. Through the production of ‘a'amu tuai, a process of decolonizing the transference of knowledge is being carried out in Rapa Nui. This process is not simple or linear, it is complex, slow and continuously negotiated, especially in the performing arts where everything is done through trial and error, but with great possibilities for creativity in the younger generations. “[R]enegotiating memory” with the Rapa Nui community is crucial to decolonizing these colonially created dominant texts and to re- invigorating and re-empowering orality. Decolonizing these recorded memories supports “the recovery of rights and identity” (Wareham, 2002, p. 198) and strengthens connections within local communities challenging colonially inherited notions of “written records to be recognized as a core component in collective memory-making” (Wareham, 2002, p. 206) and supporting the restoration of the mana and the knowledge about oral narratives, back to the Rapa Nui peoples.

## Conclusion

My research into the performative interface in Rapa Nui illuminates the dynamic interplay between Indigenous and Western knowledge systems within the context of performing arts. As a non-Indigenous researcher collaborating closely with the Rapa Nui community, I have endeavored to navigate this complex landscape, understanding that each perspective carries its own weight, histories, and significance. This inquiry is not merely an academic exercise; it is a genuine effort to engage with and honor the rich tapestry of Rapa Nui's cultural heritage while acknowledging the broader implications of colonial histories that have shaped its contemporary expressions.

Framing my research practice with the principles of Talanoa—an approach that emphasizes open dialogue and relationality—has allowed me to create a space where Rapa Nui theater practitioners can articulate their views on both “traditional” and contemporary practices. Through open-ended interviews and discussions, I have gained insights into how these practitioners perceive their cultural identity and the impact of external influences on their art forms. This process has highlighted the fluidity of cultural expression, revealing how traditions are not static but rather evolve over time through negotiation and reinterpretation.

A significant theme that emerged from my research is the concept of “tradition” as a colonial term that has historically influenced the understanding and practice of Rapa Nui performing arts. This examination reveals that while colonial narratives have often sought to categorize and confine Indigenous expressions within rigid frameworks, the reality is far more complex. Contemporary Rapa Nui performing arts are a testament to resilience and creativity, drawing from ancestral knowledge while also embracing innovation. The dialogue between traditional and contemporary practices reflects a conscious effort among practitioners to assert their cultural identity in a way that honors the past while simultaneously engaging with present realities.

The findings of these research underscore the importance of fostering intercultural dialogue in the realm of performing arts. By bridging Indigenous and Western perspectives, we can cultivate a richer understanding of artistic practices that transcends simplistic binaries. Such dialogue not only enriches our appreciation of diverse cultural expressions but also opens avenues for collaboration and mutual respect. The complexities of cultural identity, particularly in a post-colonial context, require a nuanced approach that acknowledges the multifaceted nature of both Indigenous and Western contributions to the arts.

Moreover, the implications of this research extend beyond the confines of academia. By advocating for intercultural theater practices that are informed by a deep respect for Indigenous knowledge, I have contributed to a broader movement that seeks to elevate and support the voices of marginalized communities. This research serves as a call to action for artists, scholars, and cultural practitioners to engage in meaningful partnerships that prioritize Indigenous perspectives and practices, ultimately fostering an environment where creativity can thrive.

As I continue to explore the complexities of cultural dialogue in Rapa Nui and beyond, it is essential to remain committed to understanding the intersections of history, identity, and art. The evolving landscape of performing arts in Rapa Nui offers a rich site for inquiry, reflection, and collaboration, reminding us that the stories we tell and the ways we express ourselves are deeply intertwined with our cultural heritage and the legacies we inherit. In embracing these dialogues, we not only enrich our own understanding but also contribute to a more inclusive and equitable artistic future.

## Data Availability

The original contributions presented in the study are included in the article/supplementary material, further inquiries can be directed to the corresponding author.

## Footnotes

^1^The η correlates to the “-ng” sound represented in written Māori by the ng and in Samoan by the letter g.

^2^In Chile, and through colonization in Rapa Nui as well, both surnames are used. As a patriarchal society, the former represents the father's and the latter the mother's side of the family. Over time things have changed and now it is possible to choose which surname you want first. This arrangement, does not alter the genealogical line after marriage, meaning that regardless of who I marry, in official documents I will always be Moira Sofía Fortin Cornejo, daughter of Juan Fortin and Sylvia Cornejo.
